# *Indigoferawenholdiae* (Indigofereae, Fabaceae), a new species from the Western Cape Province, South Africa

**DOI:** 10.3897/phytokeys.182.72170

**Published:** 2021-10-01

**Authors:** Brian du Preez, Leanne L. Dreyer, Charles H. Stirton, A. Muthama Muasya, Brian D. Schrire

**Affiliations:** 1 Bolus Herbarium, Department of Biological Sciences, University of Cape Town, Private Bag X3, Rondebosch, 7701, South Africa University of Cape Town Cape Town South Africa; 2 Department of Botany and Zoology, Stellenbosch University, Private Bag X1, Matieland, 7602, South Africa Stellenbosch University Stellenbosch South Africa; 3 Comparative Plant and Fungal Biology Department, Royal Botanic Gardens, Kew, Richmond, Surrey, TW9 3AE, UK Royal Botanic Gardens Richmond United Kingdom

**Keywords:** *Indigofera*, Leguminosae, Greater Cape Floristic Region, fynbos, taxonomy

## Abstract

In this study, *Indigoferawenholdiae*, a new species of *Fabaceae* from the Agulhas Plain Region of the Western Cape Province, South Africa, is described. A composite photographic plate is included along with a distribution map, description of habitat and ecology and proposed IUCN conservation status. *Indigoferawenholdiae* is unique in the *I.brachystachya* group by having digitately compound (vs. pinnately compound) leaves, white and unscented flowers (vs. pink and sweetly scented flowers) and grows on sandstone hillsides (vs. coastal limestone plains and outcrops).

## Introduction

Fabaceae represents the second largest plant family in the Cape Floristic Core Cape Region (CCR), approaching 800 species (Manning and Goldblatt 2012). The genus *Indigofera* L. with about 90 species in the region, is second only to *Aspalathus* L. (270+ species) in size (Dahlgren 1988; Schrire in Manning and Goldblatt 2012). *Indigofera* species in the CCR are largely part of a monophyletic clade referred to as the Cape Clade by Schrire et al. (2009). Unlike the three main tropical clades that have members dispersed across multiple continents, the Cape Clade is endemic to South Africa and especially within the winter-rainfall area (Schrire et al. 2009; Schrire in Manning and Goldblatt 2012). A large group within the Cape Clade, Section Brachypodae Schrire, is prolific in Fynbos vegetation and has many localised species, often associated with preferences for specific microhabitats. Diverse geological formations, as seen in the Agulhas Plain region (Thwaites and Cowling 1988), have resulted in localised radiations in many plant groups (Cowling and Holmes 1992; Manning and Goldblatt 2012). For example, over 20 new legume species have been recently described from various genera, including *Aspalathus* L., *Otholobium* C.H.Stirt., *Polhillia* C.H.Stirt., *Psoralea* L. and *Rhynchosia* Lour. (Curtis et al. 2013; Moteetee et al. 2014; Stirton and Muasya 2016, 2017; Bello et al. 2017; Du Preez et al. 2021). Members of IndigoferaSectionBrachypodae, particularly the *I.brachystachya* group (*I.brachystachya* (DC.) E.Mey. and *I.hamulosa* Schltr.), are especially diverse in this region, with several putative undescribed taxa related to *I.brachystachya*, noted from recent field studies (Schrire and du Preez, unpublished data). While further, more detailed, analyses are required to resolve this species complex, we here describe a new, distinctive species from the *I.brachystachya* group first found in the Grootbos Nature Reserve in 2020.

## Material and methods

The description of morphological characters is based on freshly collected material and herbarium voucher specimens. The conservation assessment was done using the Categories and Criteria of the IUCN (2012). The Extent of Occurrence (**EOO**) and Area of Occurrence (**AOO**) were calculated using GeoCAT (www.geocat.kew.org). The distribution map was made using QGIS 3.18 software (www.qgis.org).

## Species treatment

### Indigofera wenholdiae

Taxon classificationPlantaeFabalesFabaceae

du Preez & Schrire sp. nov.

AC4527E6-62A8-5236-AA43-855B997F63AD

urn:lsid:ipni.org:names:77220006-1

#### Diagnosis.

Similar to *I.brachystachya*, but differs in its digitately compound, 5–7 foliolate leaves (versus pinnately compound, 7–9 foliolate leaves), leaflets 4–6 mm long (versus leaflets 9–15 mm long), racemes up to 7 mm long, flowers ± 4–5 per raceme (versus racemes > 10 mm long, flowers > 8 per raceme), flowers creamy white, unscented (versus flowers pale pink, scented), branching divaricate (versus branching random), populations restricted to sandstone fynbos (versus populations restricted to limestone fynbos).

**Figure 1.**
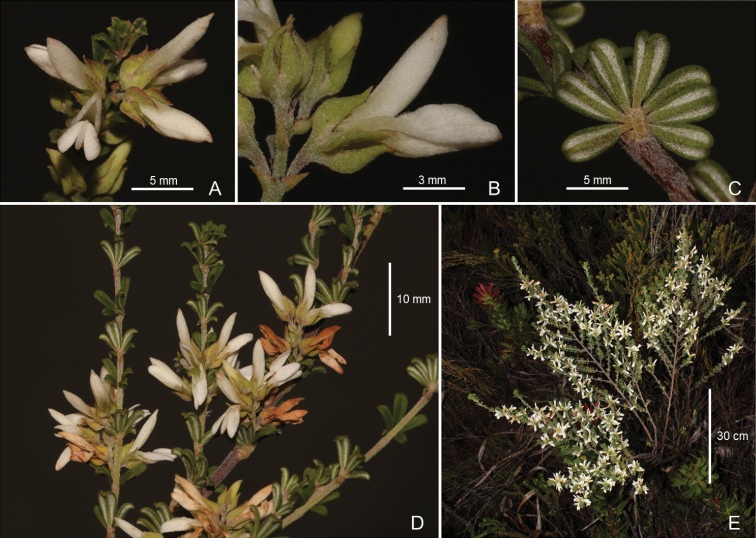
*Indigoferawenholdiae* du Preez & Schrire **A** single inflorescence **B** flower lateral view **C** leaf abaxial view **D** multiple inflorescences on branches **E** whole plant. Photographs by Brian du Preez. Voucher *B. du Preez 836* (BOL).

#### Type.

South Africa, Western Cape Province, track to Beacon Peak in Grootbos Nature Reserve, Gansbaai District, elevation 257 m, 34°31'32.84"S, 19°30'19.08"E, 7 May 2020, flowering, *B. du Preez 836* (Holotype: BOL!; Isotypes: K!, NBG!, PRE!).

#### Description.

Erect perennial shrub 0.4–0.8 m tall, robust, sparsely branching, divaricate; obligate reseeder. ***Branches*** up to 10 mm thick, terete to ribbed on fresh growth, densely strigose with sessile whitish biramous hairs, glabrescent later, reddish-brown, woody. ***Stipules*** 0.8–1.2 mm long, ± 1 mm wide at base, broadly triangular, asymmetric, navicular-cucullate, attenuate, apex aristate, adpressed to branch, adnate to base of petiole, densely strigose, ± soft-textured and thickened, pale green, pearl bodies present along margin. ***Leaves*** alternate, digitately 5–7 foliolate, petiole ± 1 mm long, sub-terete, flattened adaxially; rachis ± 0.5 mm long, terete, stipels absent; petiolules ± 0.5 mm long; terminal leaflet 4–6 mm × 1.5–2.5 mm, narrowly oblanceolate, apex apiculate, hooked, base cuneate, upper surface sparsely strigose, bright green, paler below; lower surface densely strigose; mid-vein sunken adaxially, prominent abaxially; margins not thickened, strongly revolute; lateral leaflets similar, opposite. ***Racemes*** axillary, up to 7 mm long, erect, parallel with branch, roughly equalling the leaf length, including a peduncle 2–3 mm long, ribbed, soft-textured; ± 4–5 flowered; bracts ± 1.5 mm long, lanceolate, cucullate, apex apiculate, adpressed to petiole, persistent until after flowering, pearl bodies present along margin. ***Bracteoles*** absent. ***Pedicels*** 1–1.5 mm long. ***Flowers*** 8–9 mm long, unscented. ***Corolla*** creamy-white, petals persistent after anthesis. ***Calyx*** 3.5–4.2 mm long, pale green, lobes lanceolate, distinctly navicular-cucullate, 2.5–3 mm long, ± three times tube length, moderately strigose, pearl bodies present along margin. ***Standard petal*** 8.7–9.3 mm × 3.3–3.7 mm, broadly oblong, tapering gradually to a short claw at the base; blade concave, nectar guide plain white; apex acute-obtuse; back of standard strigose, no visible colouration patterns. ***Wing petals*** 7.5–8 × 1.5–2 mm, shortly clawed at base, unguiculate portion ± half total petal length, blade asymmetrically navicular, apex rounded; blade moderately strigose. ***Keel petals*** 7.5–8 × 1.8–2.3 mm, valvately connate distally, lateral spurs up to 1 mm long, blade asymmetrically lanceolate, dorsal margin curving slightly downwards to an acute-obtuse apex, densely strigose; claws 2.5–3 mm long. ***Stamens*** 5–5.5 mm long, exceeding calyx by 1.5–2 mm, staminal tube pale creamy-green; hair clusters present below anthers. ***Gynoecium*** 4–4.5 mm long, strigillose on distal half; style ± 2 mm long, erect to ± 1 mm high distally; stigma capitate. ***Fruit*** 13–16 × 2–2.2 mm, oblong, woody, reddish-brown, densely strigillose, 4–5 seeded, dehiscent, ripe fruit not seen. ***Seeds*** not seen.

#### Distribution, habitat and ecology.

**Figure 2.**
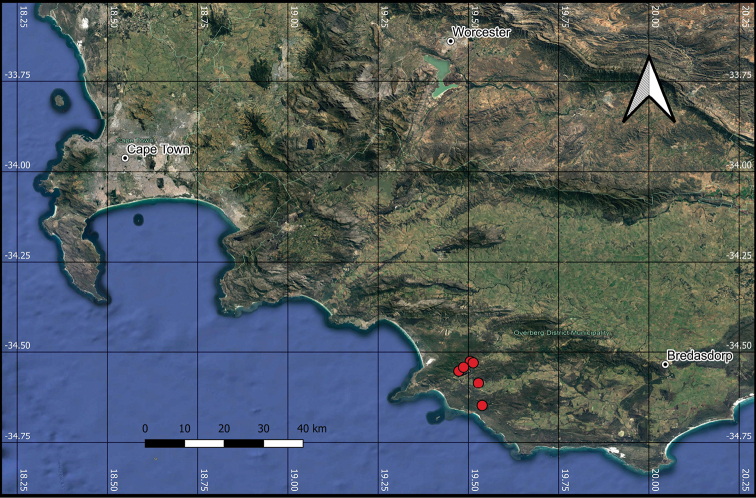
Distribution map of *Indigoferawenholdiae*.

*Indigoferawenholdiae* is restricted to a few sandstone hills from the Grootbos Nature Reserve to Pearly Beach on the Agulhas Plain of the Western Cape Province (Figure 2). The species is occasional in Overberg Sandstone Fynbos (FFs12, Mucina and Rutherford 2006), favouring south-facing slopes and hilltops.

#### Phenology.

Flowering takes place from April to July.

#### Etymology.

The specific epithet “wenholdiae” is assigned in honour of Mrs Hannerie Wenhold who has funded large-scale conservation efforts in this area, including the funding of the botanist post at Grootbos Nature Reserve of Miss Rebecca Dames who brought this species to our attention in April 2020.

#### Conservation status.

*Indigoferawenholdiae* is a range-restricted species, with an EOO of 30 km^2^ and AOO of 24 km^2^. A total of six subpopulations across four locations have been discovered, with the number of mature individuals estimated to be less than 10 000. Most subpopulations occur within protected areas or on farms earmarked for conservation. Alien vegetation is prominent in the region, although only one location is currently threatened by invasive species, while alien eradication projects at other locations have largely controlled or eradicated invasive species. The Red List category VU D2 is thus recommended, based on the IUCN Categories and Criteria (IUCN 2012).

#### Related species.

*Indigoferawenholdiae* is part of Section Brachypodae, a large group (± 30 species) of *Indigofera* species almost exclusively found in Fynbos vegetation and characterised by having five or more foliolate leaves with short petioles. The *I.brachystachya* group resolves within this section and includes *I.brachystachya*, *I.hamulosa* and several putative undescribed taxa related to the former. All of these species are characterised by a corolla morphology unique in the genus, with elongate and concave standard petals and, in general, robust rather than delicate petals. All species in this group are coastally distributed and most often occur on limestone substrate. Apart from the distinguishing features noted above, *I.wenholdiae* is an erect to less than 1 m tall, divaricately branching shrub with deep green leaves and white flowers, in contrast with the bushier growth of *I.brachystachya*, with its typically dull grey leaves and pale pink flowers.

#### Additional specimens examined.

Grootbos Nature Reserve, (3419DA), 12 September 2020, *B. du Preez 879* (BOL, NBG).

## Supplementary Material

XML Treatment for Indigofera wenholdiae
